# MSC C1: A Potential Hub Linking Stromal Plasticity, Immune Regulation, and Patient Survival in Lung Adenocarcinoma

**DOI:** 10.1111/cbdd.70414

**Published:** 2026-10-19

**Authors:** Houlei Wang, Tianle Ma, Chao Jia, Chunhui Ma

**Affiliations:** ^1^ Department of Orthopedics Shanghai Geriatric Medical Center Shanghai China

**Keywords:** bone metastasis, lung adenocarcinoma, mesenchymal stromal cells, single‐cell RNA sequencing, tumor microenvironment

## Abstract

Bone metastasis of lung adenocarcinoma (LUAD) directly leads to poor prognosis. This study aimed to dissect the stromal landscape of LUAD with bone metastases. Unsupervised clustering of scRNA‐seq data from LUAD patients with bone metastasis identifies stromal subsets. Pseudotime analysis combined with CytoTRACE stemness scoring reconstructs mesenchymal stromal cell (MSC) differentiation trajectories, while CellChat characterizes cell–cell communication networks among MSC subpopulations. Differentially expressed genes (DEGs) specific to functionally distinct MSC subsets are subjected to LASSO‐Cox regression for construction of a prognostic RiskScore model, which is validated in independent bulk RNA‐seq cohorts regarding overall survival, immune cell infiltration, and drug sensitivity. A distinct high‐stemness MSC C1 subpopulation is localized at the bifurcation point of two divergent differentiation trajectories toward a matrix‐remodeling fate and a secretory, immunomodulatory phenotype. The RiskScore model stratifies patients into low‐ and high‐risk groups with divergent overall‐survival outcomes. Reduced infiltration of cytotoxic lymphocytes is observed in high‐risk patients, and higher drug sensitivity within the high‐risk subgroup. In vitro assays verify that LDHA knockdown impairs proliferation and migration in A549 cells. This study provides a rationale for targeting stroma‐derived genes in risk‐adapted therapy.

## Graphical abstract

Mesenchymal stromal cells link niche remodeling and drug sensitivity in lung adenocarcinoma bone metastatases.

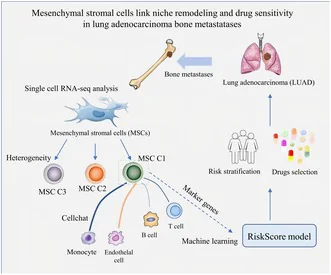

**Abbreviations**
DEGs: differentially expressed genes
GO‐BP: Gene Ontology‐Biological Process
KEGG: Kyoto Encyclopedia of Genes and Genomes
KM: Kaplan–Meier
LASSO: Least Absolute Shrinkage and Selection Operator
LUAD: lung adenocarcinoma
MSCs: mesenchymal stromal cells
OS: overall survival
ROC: receiver operating characteristic
scRNA‐seq: single‐cell RNA sequencing
ssGSEA: single‐sample Gene Set Enrichment Analysis
TCGA: The Cancer Genome Atlas
TME: tumor microenvironment

## 1 Introduction

Lung adenocarcinoma (LUAD), a common histological subtype of non‐small cell lung cancer, is a major contributor to cancer‐related mortality (Su et al. 2022; Hu et al. 2021; Wang et al. 2023). Bone represents one of the most frequent sites of distant metastasis in LUAD, affecting more than 40% of patients with advanced disease (Huang et al. 2024; Wang et al. 2024; Qin and Xu 2024). Despite advances in systemic therapies, treatment targeting the bone metastatic niche remains limited (Radeczky et al. 2021), underscoring the need to characterize its cellular and molecular architecture (Wu et al. 2020).

Metastatic progression in bone depends not only on tumor cells but also on dynamic crosstalk with the host microenvironment (Qiu et al. 2024; Calderon‐Espinosa et al. 2024). The bone marrow constitutes a specialized ecosystem composed of stromal, immune, vascular, and hematopoietic components (Nakai et al. 2025) that collectively support tumor colonization, survival, and therapy resistance (Chen, Zhang, and García Cenador 2024). As key perivascular residents of the bone marrow, mesenchymal stromal cells (MSCs) play fundamental roles in sustaining skeletal homeostasis, regulating immune responses, and modulating extracellular matrix dynamics (Zheng et al. 2022; Zeng et al. 2025; Senthilkumar et al. 2023; Venugopal et al. 2023). In cancers such as breast and prostate carcinoma, tumor‐educated MSCs promote immunosuppression, angiogenesis, osteolysis, and chemoresistance (Antoon et al. 2024). However, the functional heterogeneity of the MSC compartment during lung cancer bone metastasis remains poorly defined (Sun and Yao 2023), largely due to the fact that bulk transcriptomic approaches cannot resolve cellular diversity.

Advances in single‐cell RNA sequencing (scRNA‐seq) have enabled high‐resolution dissection of complex tissue microenvironments, revealing previously unrecognized stromal and immune subsets with distinct biological functions (Wang et al. 2025). Although scRNA‐seq studies have begun to characterize the TME in primary lung cancers (Huang et al. 2025), comprehensive profiling of human LUAD bone metastases remains limited. Consequently, the transcriptional states, lineage trajectories, and intercellular communication networks of stromal populations within this clinically important metastatic site are poorly understood (Guan et al. 2025). Moreover, it is unclear whether the stromal‐derived molecular model can serve as reliable biomarkers for predicting the prognosis and therapeutic response in LUAD.

To address these limitations, this study integrated scRNA‐seq data from LUAD bone metastases with bulk RNA‐seq and clinical data from multiple independent cohorts. The cellular composition of the bone metastatic niche was characterized, stromal heterogeneity and lineage trajectories were delineated, and cell–cell interactions were mapped. Stromal‐specific genes were identified to construct a robust prognostic model, which was further assessed for its association with immune infiltration patterns and predicted drug sensitivity. This study provides a comprehensive framework for understanding stromal contributions to LUAD bone metastasis.

## 2 Materials and Methods

### 2.1 Data Collection

Gene expression profiles in TPM format were obtained from The Cancer Genome Atlas (TCGA)‐LUAD cohort via the UCSC Xena platform (https://gdc‐hub.s3.us‐east‐1.amazonaws.com/download/TCGA‐LUAD.star_tpm.tsv.gz). After quality control and filtering, 490 primary tumor samples and 58 matched adjacent non‐tumor tissue samples were retained for further analysis. Gene Expression Omnibus database (GEO, https://www.ncbi.nlm.nih.gov/geo/) was accessed to obtain RNA‐seq data and corresponding clinical information from 226 LUAD patients in the GSE31210 cohort. In addition, the scRNA‐seq dataset GSE266330, which contained 10 lung cancer patients with bone metastases, was obtained from the GEO database.

### 2.2 Data Preprocessing and scRNA‐Seq Analysis

TCGA samples lacking clinical follow‐up or with survival ≤ 30 days were excluded. Ensembl IDs were converted to gene symbols, and for duplicated symbols, the highest expression value was retained. For the GSE31210, normal samples were removed and probes were mapped to gene symbols using platform annotations. For the single‐cell data, doublets were removed using DoubletFinder with sample‐specific thresholds based on 10× Genomics guidelines. Ambient RNA contamination was corrected using decontX, and low‐quality cells were filtered out by retaining cells with mitochondrial gene content < 10%, detected genes between 200 and 5000, and total Unique Molecular Identifier (UMIs) < 100,000. Data were normalized using the SCTransform function in the Seurat R package, followed by principal component analysis (PCA) using RunPCA and batch effect removal using the Harmony R package. UMAP was computed using the top 20 PCs. Cells were clustered using the FindNeighbors and FindClusters functions at resolution 0.1 and annotated based on marker genes from the CellMarker 2.0 database and a previous study (Liu et al. 2025).

### 2.3 Pseudotime Trajectory Inference

To reconstruct MSC differentiation dynamics and lineage trajectories, pseudotime analysis was performed using Monocle2 (version 2.14.0, USA) (Qiu et al. 2017). Lowly expressed genes detected in fewer than 10 cells were removed during CellDataSet construction. Data were normalized and dispersion estimated using the estimateSizeFactors and estimateDispersions, respectively. Feature genes for trajectory construction were identified with differentialGeneTest. Dimensionality reduction was performed using the reduceDimension function, with parameters set as max_components = 2 and reduction_method = “DDRTree”. Next, cells were ordered along pseudotime by orderCells, and the trajectory root was defined based on CytoTRACE stemness scores.

Marker genes for each MSC subpopulation were identified using the FindAllMarkers function and used to define cellular states along the trajectory. After preprocessing, dimensionality reduction was performed with the reduceDimension function (setting max_components = 2, method = “DDRTree”), and cells were ordered along pseudotime using the orderCells function to reconstruct a continuous differentiation path. To define the trajectory root, CytoTRACE‐derived stemness scores were incorporated, and the cell state with the highest stemness was designated as the starting point. Pseudotime‐associated genes were then subjected to Gene Ontology biological process (GO‐BP) and Kyoto Encyclopedia of Genes and Genomes (KEGG) enrichment analysis using the clusterProfiler R package (version 3.14.3, China) (Wu et al. 2021).

### 2.4 Development and Verification of a Robust Prognostic Model

A prognosis model was developed using marker genes related to MSC subpopulation. Specifically, DEGs (|log₂FC| > log_2(2), *p*.adj < 0.05) between tumor and adjacent tissues were identified by the limma R package (version 3.42.2, Australia) (Qiu et al. 2023), and the Benjamini‐Hochberg method was used for multiple‐testing correction. Subpopulation‐specific marker genes were identified with FindAllMarkers in the Seurat package (v3.2.3, USA), and marker genes were determined according to subpopulation‐specific expression patterns with parameters set as min.pct = 0.25 and logfc.threshold = 0.5. Next, these DEGs were intersected with markers specific to MSC C1 to generate a candidate gene set, which was then subjected to univariate Cox regression to identify genes significantly associated with OS. To minimize overfitting, LASSO Cox regression with 10‐fold cross‐validation was performed using the glmnet R package to select the optimal regularization parameter (λ). Stepwise multivariate Cox regression based on the Akaike Information Criterion (AIC) was subsequently performed using the MASS package to determine the final gene set. The RiskScore for each patient was calculated as follows: RiskScore = $\Sigma \left(\mathrm{\beta i}\times \text{Expi}\right)$, where Expi denotes the expression level of gene i, and βi is its coefficient from the multivariate Cox model. Next, RiskScore values were z‐score normalized and dichotomized into high‐ and low‐risk groups using the median as cutoff. Model performance was assessed by Kaplan–Meier (KM) survival analysis with the log‐rank test using the survival and survminer R packages, and by time‐dependent ROC curves using the timeROC R package (version 0.4, France) (Li et al. 2025).

### 2.5 Immune Infiltration Analysis

Enrichment scores for 28 immune cell types were computed using single‐sample Gene Set Enrichment Analysis (ssGSEA) in the GSVA R package, based on gene signatures obtained from a reference study (Charoentong et al. 2017). In addition, immune cell infiltration was estimated using the TIMER algorithm, which infers the relative abundances of six major immune cell types.

### 2.6 Association Between the RiskScore and Drug Sensitivity

Drug sensitivity, expressed as predicted IC_50_ values, was estimated for the TCGA‐LUAD samples using the oncoPredict R package (Maeser et al. 2021). The relationship between the RiskScore and drug response was assessed by Pearson correlation analysis across all samples. Correlations with *p* < 0.05 and |r| > 0.3 were considered statistically significant.

### 2.7 Cell Culture and Transfection

The LUAD cell line A549 (CBP60084) and human lung epithelial cells BEAS‐2B (CBP60577) were purchased from COBIOER Biotechnology Co. Ltd. (Nanjing, Jiangsu, China). The specialized A549 complete medium (CBP60084M, COBIOER) and BEAS‐2B complete medium (CBP60577M, COBIOER) supplemented with 10% FBS and 1% penicillin–streptomycin were used for cell culture at 37°C in 5% CO_2_. For siRNA‐mediated knockdown, cells were transfected with LDHA‐specific siRNA (si‐*LDHA*#1 (SS: 5′‐UUCCUUAUCUUUAUCAGUCCC‐3′, AS: 5′‐GACUGAUAAAGAUAAGGAACA‐3′) and si‐*LDHA*#2 (SS: 5′‐UCCGUAAAGACCCUUAAUCAU‐3′, AS: 5′‐GAUUAAGGGUCUUUACGGAAU‐3′)) or negative control siRNA (si‐NC) using Lipofectamine 2000 Reagent (#11668027, Invitrogen, USA), according to the manufacturer's protocol. For downstream analyses, cells were harvested or assayed 48 h (h) post‐transfection.

### 2.8 Gene Expression Detection In Vitro

The TRIzol reagent (R0017S, Beyotime, Shanghai, China) was used to extract total RNA from cells, according to the protocol. Next, cDNA was synthesized from 1 μg RNA using the BeyoRT II cDNA Synthesis Kit (Beyotime, D7168S) with oligo (dT) primers at 42°C for 1 h, and heated at 85°C to terminate the reaction. qRT‐PCR was performed in triplicate using an ABI 7500 Real‐Time PCR System (Thermo Fisher Scientific, Foster City, CA, USA) with SYBR Green Master Mix (Beyotime, D7260). The 20 μL reaction solution contained gene‐specific primers and cDNA template. The thermal cycling conditions were as follows: 95°C for 2 min, followed by 40 cycles at 95°C for 15 s, at 60°C for 30 s. Relative expression was calculated using the 2^−ΔΔCt^ method based on three independent biological replicates. Primer sequences are provided in Table 1.

**TABLE 1:** The specific primer sequence for qPCR assay.

Genes	Forward (5′‐3′)	Reverse (5′‐3′)
PPFIBP1	GGGACAGGCATCAGGAAGTG	CTGAATGATGGAAGAACTCGCA
DOCK4	CACTCGCCAGGACTCATCTC	GGCCGACTGTTCATTCACCT
LDHA	ACCGTGTTATTGGAAGCGGT	CTCCATGTTCCCCAAGGACC
NT5E	AACTTCCTGGCCAATGGTGG	ACCACTAGTAGCTCCCCTTCT
C4orf48	ACTGTCCGTGTGGGCGCTA	TCCTTCTCCCCCCCTCGTCC
NUPR1	AAGGTCGCACCAAGAGAGAA	TCATGGCAGAAGCACCACC
ID1	GGTAAACGTGCTGCTCTACG	TTGCTCACCTTGCGGTTCT
GAPDH	GGTCATCCCTGAGCTGAACG	GTCAGGTCCACCACTGACAC

### 2.9 Cell Proliferation Assay

A549 cells (2 × 10^3^/well in 100 μL medium) were seeded into 96‐well plates, and cell proliferation was measured at 0, 24, 48, and 72 h using the CCK‐8 assay (Beyotime, C0037) (Yu et al. 2023). At each time point, 10 μL CCK‐8 reagent was added, followed a Gene Ontology biological process (GO‐BP) and 30‐min incubation. According to the protocol, OD₄₅₀ was measured with a microplate reader (Infinite M200 Pro, Tecan, Switzerland).

### 2.10 Wound Healing and Trans‐Well Assay

Wound healing and Transwell assays were performed according to a previously described protocol (Qian et al. 2023). For the wound healing assay, si‐LDHA#1–transfected A549 cells (5 × 10^5^/well) were seeded into 6‐well plates and cultured until reaching near confluence. A scratch was generated using a sterile 200‐μL pipette tip. After washing the cells with PBS, wound closure was imaged at 0 and 48 h using an inverted microscope (IX73, Olympus, Japan). For the Transwell assay, 5 × 10^4^ cells suspended in 300 μL of serum‐free medium were seeded into the upper chamber of an 8‐μm pore insert (Corning). The lower chamber contained 700 μL complete medium (10% FBS). Following incubation for 48 h, cells remaining on the upper surface of the insert were removed, fixed with a cotton swab, and the migrated cells on the lower surface were fixed with 4% paraformaldehyde (P0099, Beyotime, Shanghai, China) at room temperature for 30 min. After PBS washing, the cells were stained with 0.5% crystal violet (C0121, Beyotime), rinsed with water to remove excess dye, imaged, and quantified under an inverted microscope (IX73, Olympus).

### 2.11 Western Blot

Total cellular proteins were extracted using RIPA lysis buffer (P0013B, Beyotime). Protein concentration was determined using the BCA Protein Assay Kit (P0010, Beyotime), and equal amounts of protein were loaded for electrophoresis. The extracted proteins were separated by SDS‐PAGE (P0015L, Beyotime) and transferred onto PVDF membranes (FFP32, Beyotime). Proteins were separated on 15% SDS‐PAGE gels (for low‐molecular‐weight proteins including C4orf48 and NUPR1). The membranes were then blocked with 5% skimmed milk for 2 h to prevent non‐specific binding and then incubated overnight at 4°C with the following primary antibodies: PPFIBP1 (1:2000, ab195339, abcam, Cambridge, UK), DOCK4 (1:2000, ab85723, abcam), LDHA (1:1000, ab47010, abcam), NT5E (1:1000, ab133582, abcam), C4orf48 (1:1000, sc‐245,793, Santa Cruz Biotechnology, Dallas, Texas, USA), NUPR1 (1:1000, #25145, Cell Signaling Technology, Danvers, MA), ID1 (1:1000, ab230679, abcam), and GAPDH (1:10000, ab181603, abcam) as internal reference. After washing with TBST, the membranes were further incubated with goat anti‐rabbit IgG H&L (HRP) (1:2500, ab205718, abcam) or Donkey Anti‐Goat IgG H&L (HRP) (1:2000, ab6885, abcam) for 1.5 h at room temperature. Protein bands were visualized using BeyoECL Plus (P0018S, Beyotime), and band intensities were quantified using ImageJ software (Version 1.8.0).

### 2.12 Statistical Analysis

All statistical analyses were conducted using R software (version 3.6.0) and SangerBox 2 tool (Chen, Xu, et al. 2024). OS between different risk groups was analyzed using the log‐rank test. Continuous variables were analyzed by Student's *t*‐test, and Pearson correlation analysis was performed to assess the association between risk gene expression levels and drug IC_50_ values. qPCR, Western blot and CCK‐8 data were analyzed using one‐ or two‐way ANOVA, while wound healing and Transwell results were examined using Welch's *t*‐test. A *p*‐value < 0.05 was considered to indicate statistical significance.

## 3 Results

### 3.1 Mesenchymal Stromal Cells Were Enriched and Robustly Identified in the Bone Metastatic Niche

Following stringent quality control, data normalization, dimensionality reduction, and unsupervised clustering analysis, a total of 26,822 high‐quality cells were obtained from the GSE266330 dataset. These cells were classified into 10 distinct clusters (Figure 1A) and annotated into eight major cell types based on the canonical marker gene expression profiles: T cells, monocytes, mesenchymal stromal cells (MSCs), B cells, endothelial cells, hematopoietic stem/progenitor cells (HSPCs), plasma cells, and plasmacytoid dendritic cells (pDCs) (Figure 1B–C). Among the identified cell types, MSCs were selected for further analysis due to their consistent presence across samples and relatively high abundance (Figure 1D). Moreover, MSCs exhibited robust expression of canonical markers such as *THY1* and *LEPR*, further supporting their identity and highlighting their potential relevance to bone metastasis.

**FIGURE 1:**
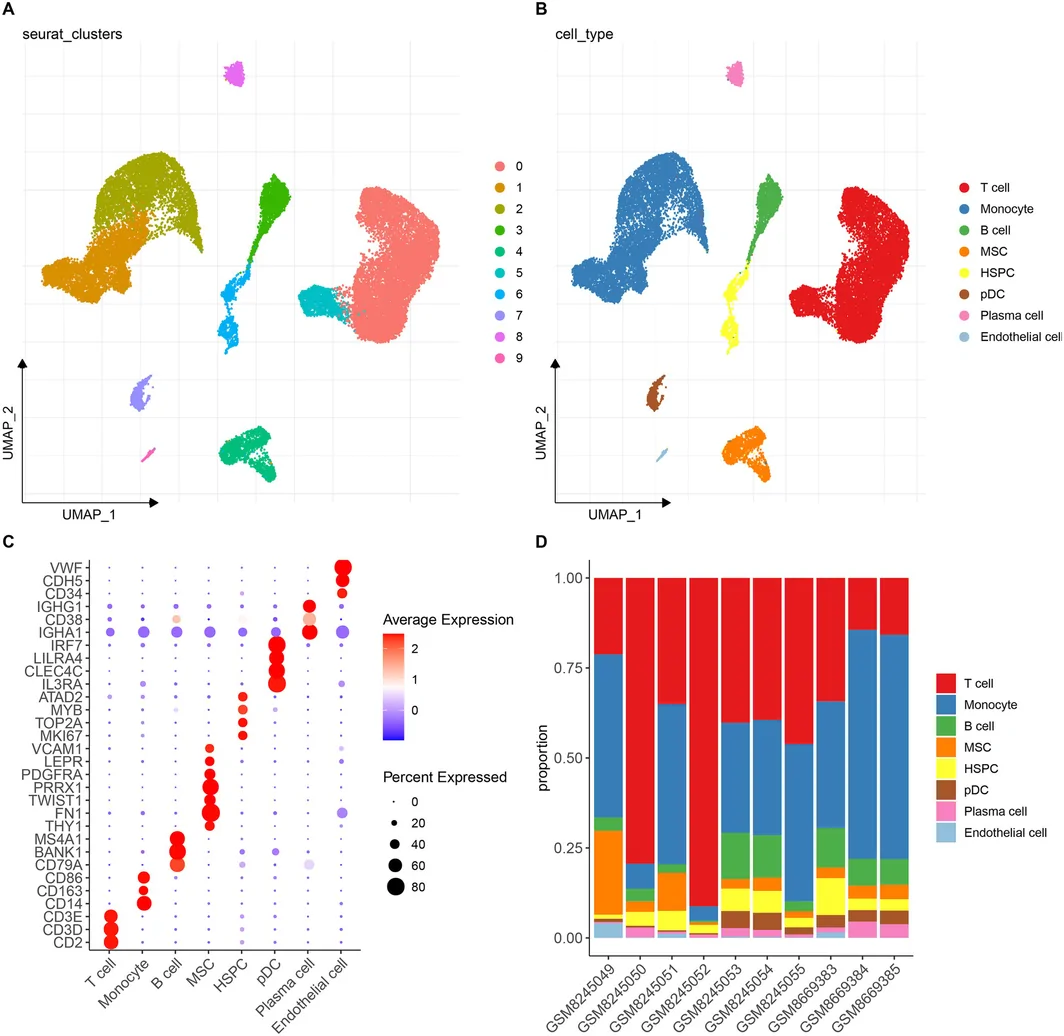
Characterization of cellular composition in human LUAD bone metastases. (A, B) UMAP plots showing the clustering of cells before (A) and after (B) annotation with cell type identities. (C) The expression levels of canonical marker genes across annotated cell populations. (D) Stacked bar chart depicting the proportion of each cell type.

### 3.2 Transcriptional Heterogeneity Underlied Functional Diversification of MSCs in Bone Metastasis

Given the critical role of MSCs in shaping the bone metastatic microenvironment, their transcriptional heterogeneity was explored. MSCs were isolated from the integrated scRNA‐seq data and re‐clustered after t‐SNE dimensionality reduction, revealing three distinct subpopulations, designated as C1, C2, and C3 (Figure 2A). Each subpopulation was characterized by a unique set of marker genes (Figure 2B). To further characterize their biological functions, KEGG pathway enrichment analysis was performed on the subcluster‐specific marker genes. The results revealed distinct functional programs among the three subpopulations. Specifically, C1 was significantly enriched in pathways related to protein synthesis and stromal interaction, including Ribosome, ECM‐receptor interaction, and HIF‐1 signaling pathway (Figure 2C). In contrast, C2 showed prominent enrichment in PI3K‐Akt signaling pathway and TNF signaling pathway, suggesting potential involvement in survival signaling and inflammation (Figure 2D). C3 was primarily enriched in cGMP–PKG signaling pathway and cAMP signaling pathway, implicating this subpopulation in cell motility and microenvironmental sensing (Figure 2E). Thus, these findings indicated that the heterogeneity of MSCs may contribute to their functional diversification during bone metastasis progression.

**FIGURE 2:**
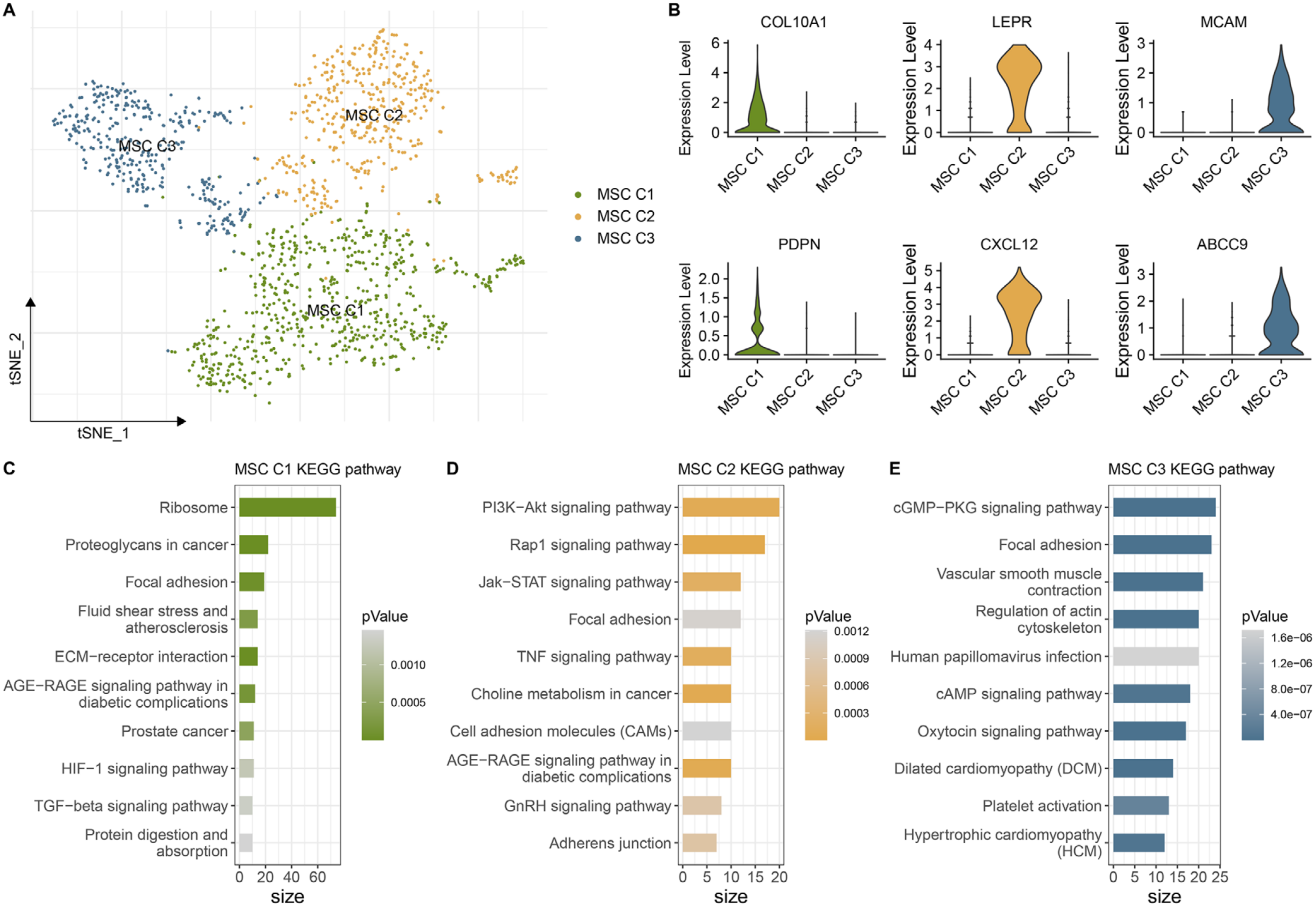
Sub‐clustering and functional characterization of mesenchymal stromal cells (MSCs). (A) t‐SNE plot showing the sub‐clustering of MSCs into three distinct populations. (B) Violin plots depicting the expressions of signature genes in each MSC subpopulation. (C–E) KEGG pathway enrichment analysis of DEGs in MSC C1, MSC C2, and MSC C3.

### 3.3 MSC Heterogeneity Reflected Divergent Differentiation Trajectories Toward Matrix‐Remodeling and Secretory Fates

Trajectory inference analysis was performed to investigate the developmental relationships among MSC subpopulations within the bone metastatic niche. The results revealed that MSCs underwent a gradual state transition along a continuous differentiation trajectory, with two distinct branches emerging at the terminal end (Figure 3A–C). To estimate the relative differentiation status of individual MSCs, CytoTRACE scores ranging from 0 to 1 were calculated, with higher scores indicating greater stemness. State 2 exhibited the highest CytoTRACE scores and was thus designated as the root of the trajectory (Figure 3A). Notably, the majority of C1 cells were localized near the initial state, consistent with a relatively less‐differentiated phenotype (Figure 3B,C). Along the pseudotime axis, MSCs progressively diverged into two major lineages (Figure 3D). One branch was characterized by the progressive enrichment of biological processes related to the regulation of cell junction assembly, features suggestive of an osteogenic or matrix‐remodeling fate, ossification, extracellular structure organization, extracellular matrix organization, and negative regulation of cell adhesion (Figure 3E). In contrast, the other branch was characterized by increasing activity in the establishment of protein localization to the endoplasmic reticulum and protein targeting to the ER, suggesting enhanced secretory capacity (Figure 3F).

**FIGURE 3:**
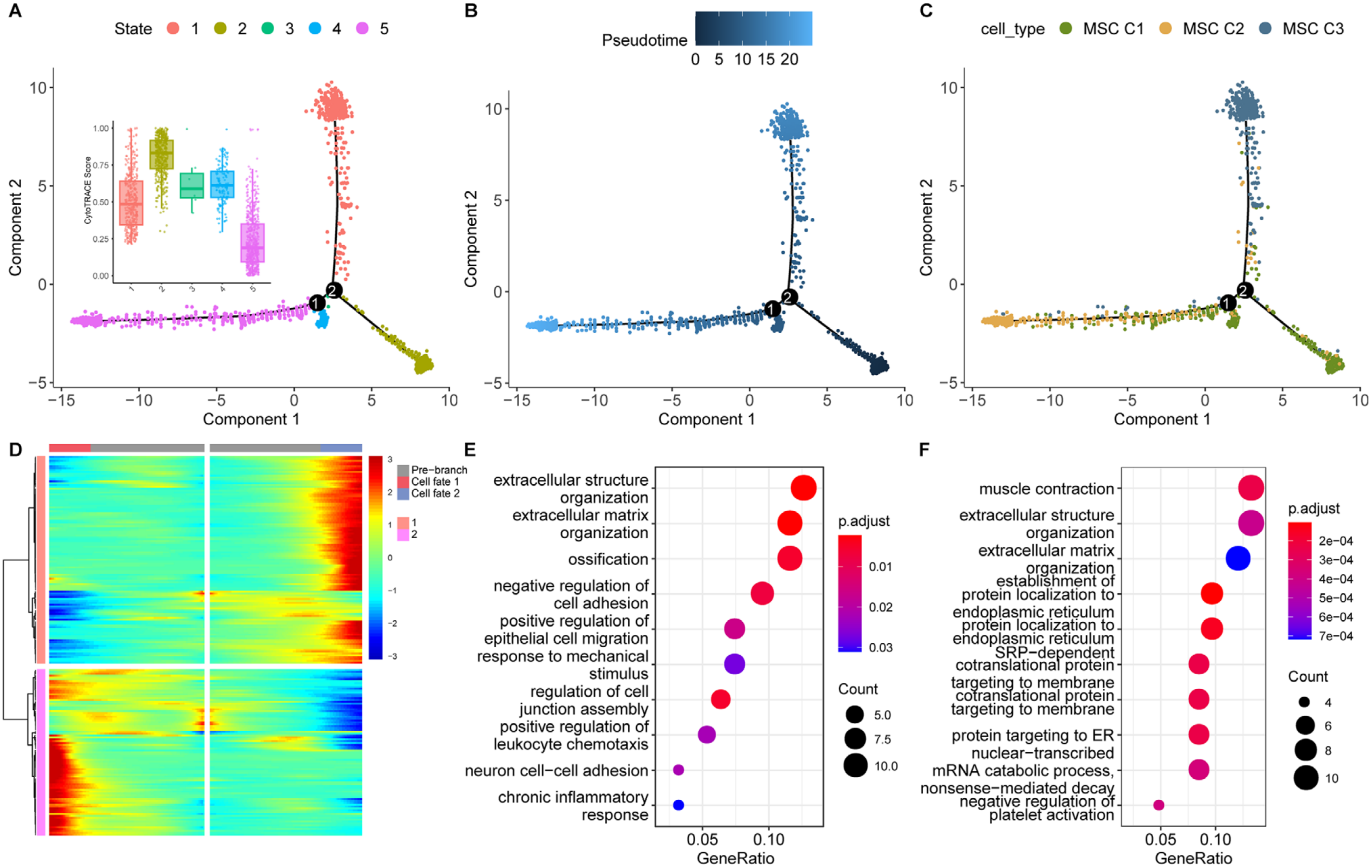
Pseudotime trajectory analysis. (A) Trajectory inference of MSCs colored by inferred cellular states (State 1–5). (B) Same trajectory colored by pseudotime. (C) Trajectory colored by cell type identity (MSC C1, C2, C3). (D) Heatmap of DEGs at branch points. (E, F) GO‐BP enrichment analysis of genes upregulated in fate 1 (E) and fate 2 (F).

### 3.4 MSC C1 Acted as a Central Communication Hub

Cell–cell communication analysis was performed to elucidate the role of MSCs in the lung cancer bone metastatic microenvironment. The results showed that MSCs engaged in extensive crosstalk with multiple cell populations (Figure 4A). Across the cellular populations, the number and strength of ligand‐receptor interactions were highly correlated, with the MSC C1 subpopulation exhibiting the most active communication with neighboring cell types (Figure 4A), suggesting that C1 may serve as an important mediator of intercellular signaling within the tumor microenvironment. Analysis of signaling inflow and outflow further revealed that MSC C1 received significantly more incoming signals than other cell types (Figure 4B). MSC C1 predominantly transmitted signals to monocytes and endothelial cells through ligands including TGFβ1/3, VEGFA, PDGFs, and WNT5B and their corresponding receptors, including TGFBR1/2, VEGFR1/2, PDGFRs, and FZD1/4. These signaling interactions were associated with biological processes related to angiogenesis, immune suppression, and extracellular matrix remodeling (Figure 4C). Conversely, MSC C1 received strong incoming signals from B cells, monocytes, and T cells, primarily via the MIF‐ACKR3, MDK‐SDC1, PTN‐NCL, and IGF1‐IGF1R axes. Among these interactions, the MIF‐ACKR3 axis was particularly enriched and has been implicated in inflammation and tumor progression, indicating that active modulation by immune‐derived signals occurs within the metastatic niche (Figure 4D). Collectively, these findings positioned MSC C1 as a central hub of bidirectional intercellular communication in the bone metastatic microenvironment.

**FIGURE 4:**
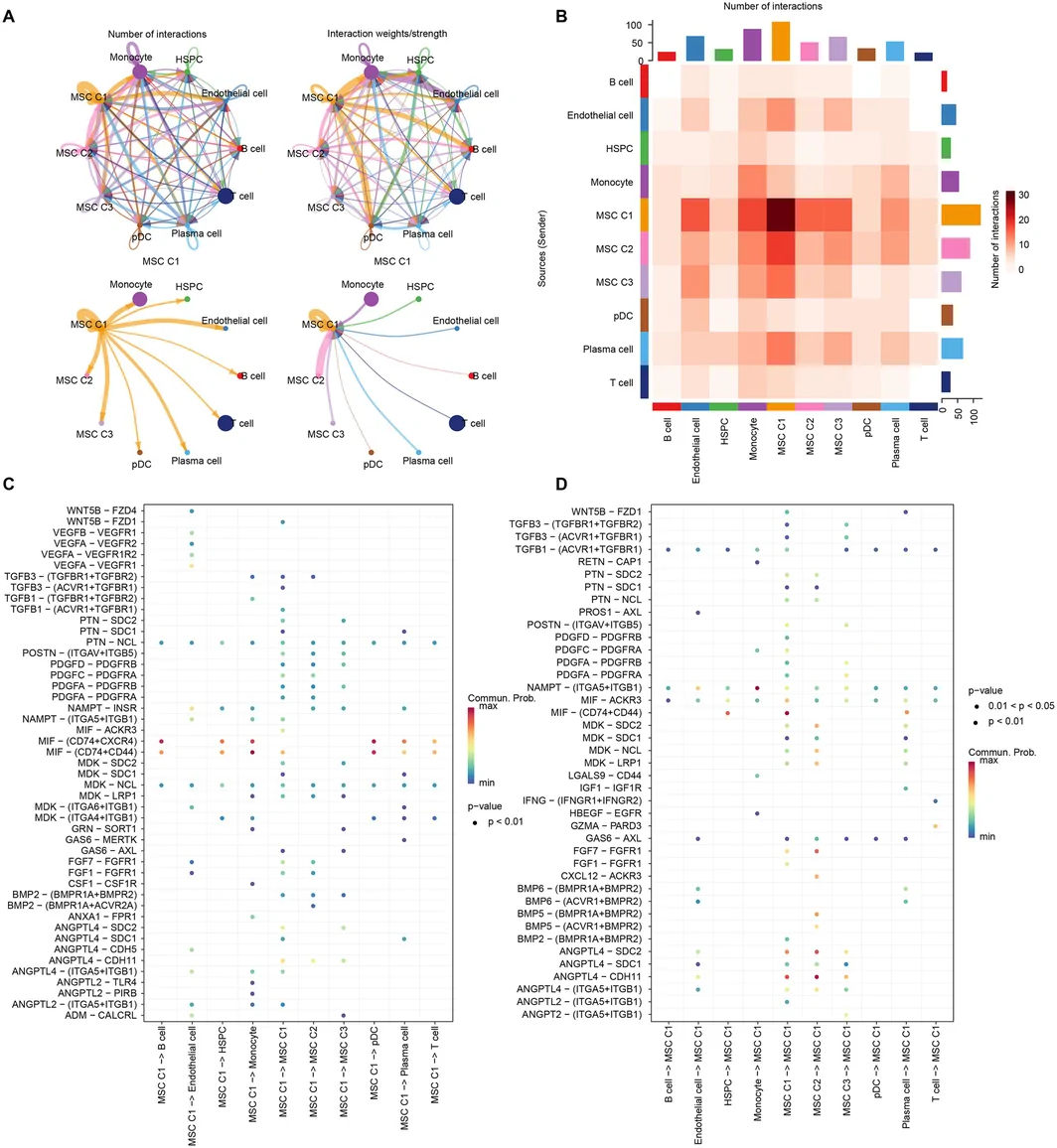
Cell–cell communication landscape in LUAD bone metastases. (A) Cell–cell communication network inferred from ligand‐receptor interactions. (B) Heatmap depicting the total communication strength between all cell types. (C, D) Bubble plots showing ligand‐receptor pairs involved in signaling from MSC C1 to other cell types and from other cell types to MSC C1.

### 3.5 Construction and Validation of a Clinical Prognostic Model

MSC C1 marker genes were integrated with transcriptomic data from the TCGA cohort to develop a prognostic gene model. Differential expression analysis identified 2508 DEGs between tumor and normal tissue, of which 102 overlapped with MSC C1‐specific marker genes. Univariate Cox regression selected 34 genes significantly associated with overall survival (OS; *p* < 0.05). Subsequent LASSO‐Cox regression at the optimal λ = 0.0172 (Figure 5A) reduced the gene set to 16 genes, and stepwise selection based on AIC further refined the model to a 7‐gene prognostic model (Figure 5B) for the final RiskScore = 0.297*PPFIBP1‐0.343*DOCK4 + 0.474*LDHA+0.078*NT5E+0.152*C4orf48–0.205*NUPR1 + 0.129*ID1. RiskScores were z‐score normalized, and patients in the TCGA cohort were stratified into low‐ and high‐risk groups. KM analysis demonstrated that patients in the high‐risk group had significantly shorter OS than those in the low‐risk group (Figure 5C, *p* < 0.05). Time‐dependent ROC analysis showed moderate predictive performance of the model, with an AUC ranging from 0.65 to 0.75 for 1‐ to 5‐year OS prediction (Figure 5D). To further evaluate the robustness of the prognostic model, the RiskScore was also applied to the independent GSE31210 validation cohort. The results showed that the model effectively stratified patients into distinct high‐ and low‐risk groups, with a high AUC (Figure 5E–F). These findings confirmed the robustness and generalizability of the MSC C1‐derived prognostic model.

**FIGURE 5:**
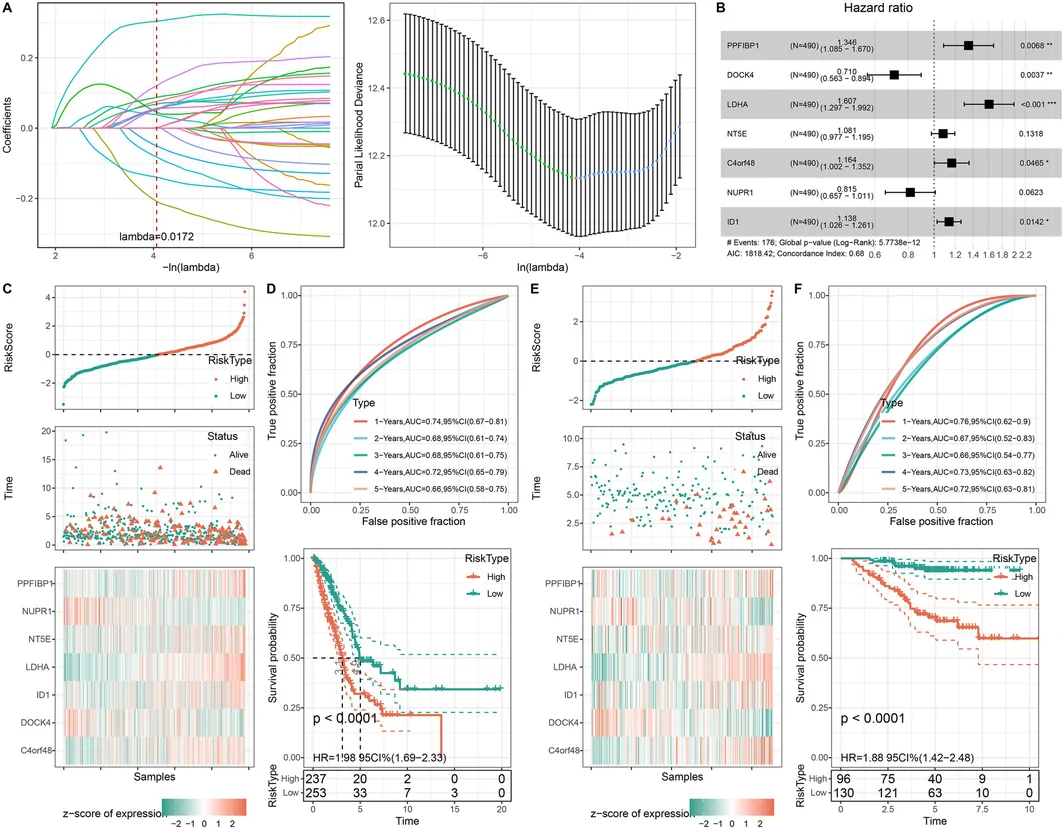
Development and validation of a prognostic signature. (A) LASSO Cox regression analysis showing the trajectory of coefficient values for each predictor across varying lambda values. (B) Forest plot displaying hazard ratios (HR) and 95% confidence intervals for key genes (*p < 0.05, **p < 0.01, ***p < 0.001). (C) Distribution of the RiskScore, survival status, and expression levels of signature genes in the TCGA‐LUAD cohort. (D) ROC and KM‐survival curves in TCGA‐LUAD. (E, F) Survival status, gene expression, ROC and KM survival curves in GSE31210.

### 3.6 Risk Stratification Was Correlated With Immune Activation and Differential Chemotherapeutic Response

Analysis of immune cell infiltration revealed that the low‐risk group exhibited significantly higher infiltration levels of activated B cells, CD8^+^ T cells, effector memory CD8^+^ T cells, macrophages, and immature B cells (Figure 6A,B). These results suggested that a lower RiskScore was associated with more active anti‐tumor immune responses, characterized by enhanced adaptive immunity and immune cell activation. Meanwhile, IC_50_ values of multiple chemotherapeutic agents were estimated in the TCGA‐LUAD cohort and correlated with RiskScore. Thirteen drugs showed significant correlation with the RiskScore (|r| > 0.3, *p* < 0.05). Among these, ERK_6604_1714 exhibited a significant negative correlation with RiskScore (Figure 6C), suggesting that the high‐risk group was more sensitive to this compound and highlighting its potential for personalized therapy.

**FIGURE 6:**
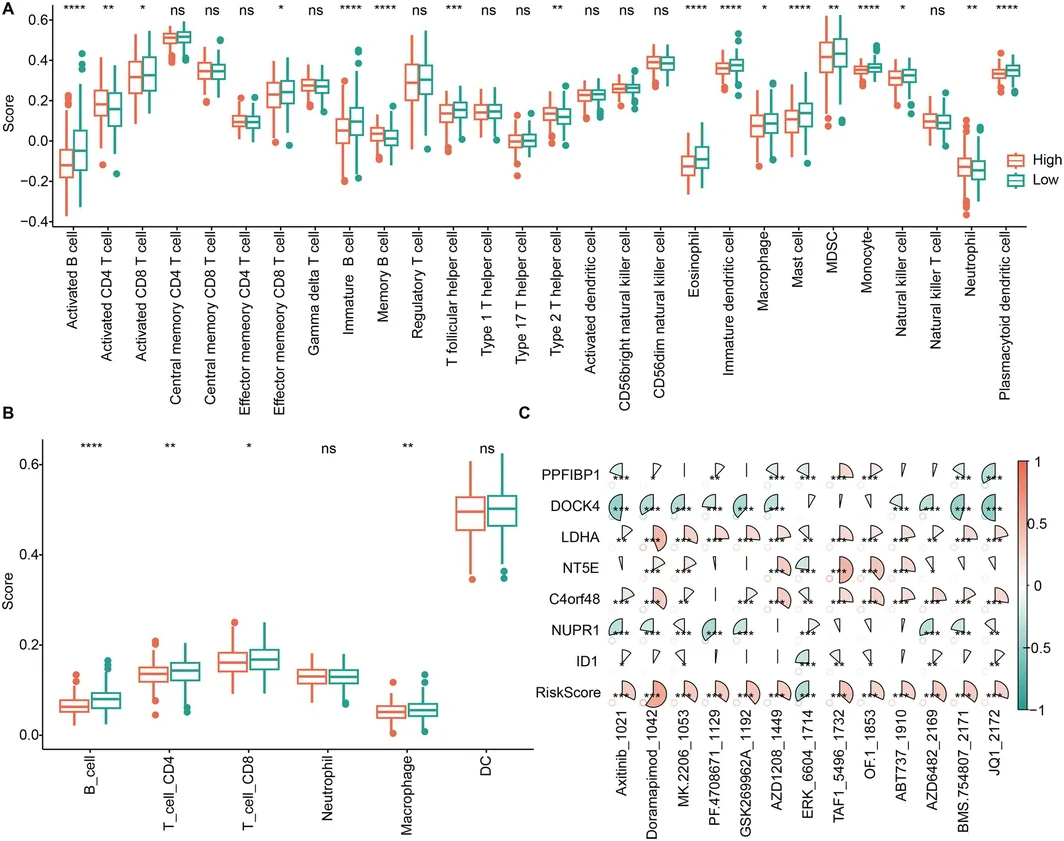
Immune microenvironment and drug sensitivity analysis. (A) Differences in immune cell scores between high‐ and low‐risk groups assessed by ssGSEA. (B) Differences in immune cell scores between the two risk groups estimated by TIMER. (C) Correlation analysis between RiskScore, expression of key signature genes, and IC50 values of multiple drugs in the TCGA‐LUAD cohort. (ns is no significance, *p < 0.05, **p < 0.01, ***p < 0.001, ****p < 0.0001)

### 3.7 LDHA Promoted Tumor Cell Proliferation and Migration in LUAD

qPCR analysis revealed significantly higher mRNA expression levels of *LDHA*, *NT5E*, *C4orf48*, *NUPR1*, and *ID1* in A549 cells than in BEAS‐2B cells (Figure 7A). Among these upregulated genes, *LDHA* exhibited the highest mRNA expression level, suggesting that it may play a particularly important role in A549 cells. Accordingly, *LDHA* was selected for subsequent knockdown functional validation. Following siRNA transfection, *LDHA* expression was markedly downregulated, with si‐*LDHA*#1 exhibiting higher silencing efficiency and therefore being selected for later experiments (Figure 7B). Cell proliferation assays revealed that si‐*LDHA* significantly suppressed the proliferation of A549 cells (Figure 7C). Importantly, both Transwell and wound healing assays showed that *LDHA* knockdown substantially impaired cell migration (Figure 7D,E). Consistently, Western blot analysis demonstrated significantly higher protein expression levels of *LDHA, NT5E, C4orf48, NUPR1*, and *ID1* in A549 cells than in BEAS‐2B cells (Figure 7F). Hence, *LDHA* may exert a critical oncogenic role in promoting LUAD cell proliferation and migration.

**FIGURE 7:**
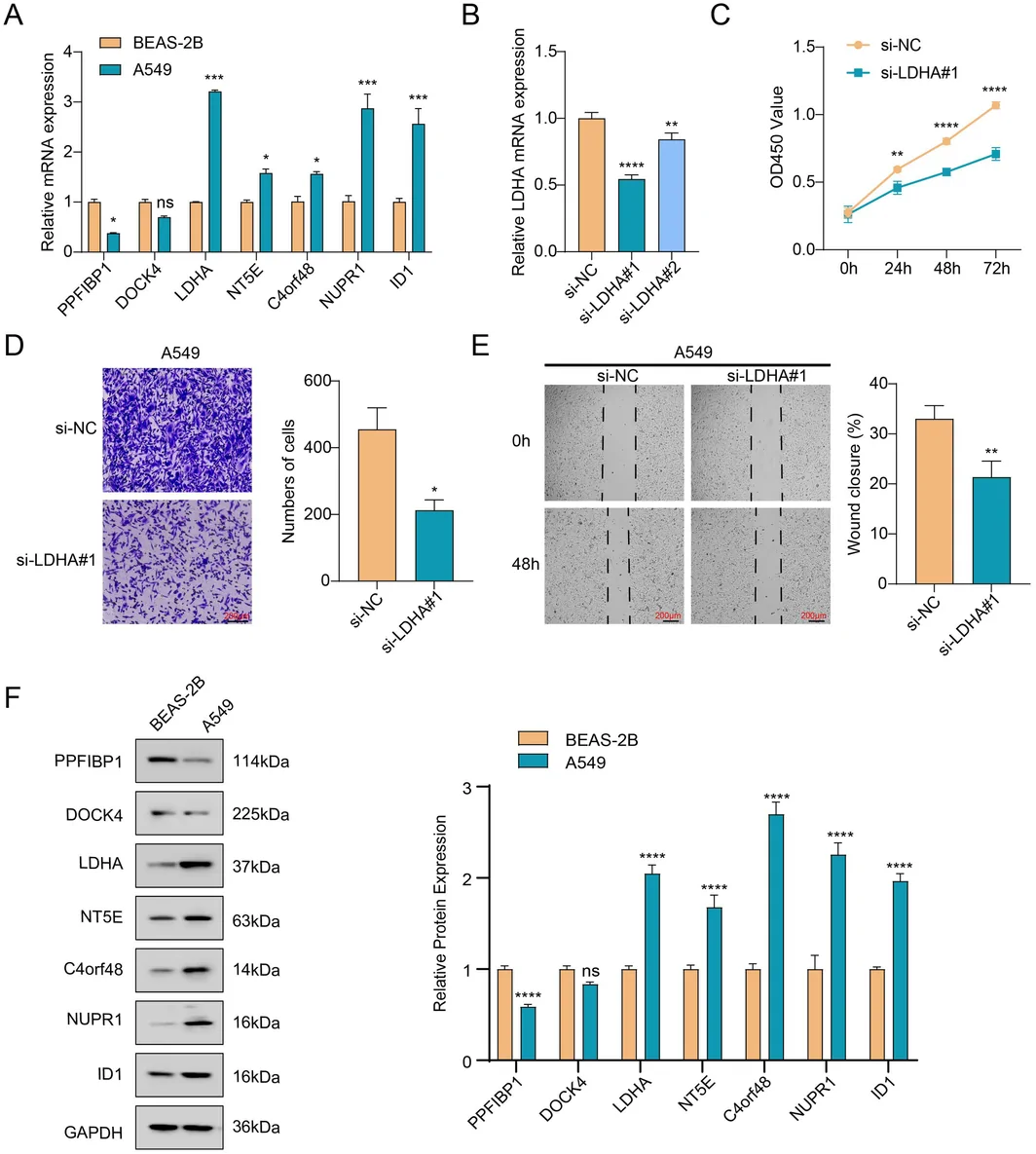
LDHA was upregulated in tumor cells and affected proliferation and migration. (A) qPCR detected the expression of model genes in vitro. (B) qPCR for silencing efficiency analysis in cells with two siRNAs targeting *LDHA*. (C) CCK‐8 assay for cell proliferation assessment. (D, E) Trans‐well and wound healing assays for cell migration assessment. (F) Western blot analysis showing the protein expression levels of model genes in BEAS‐2B and A549 cells. (ns is no significance, *p < 0.05, **p < 0.01, ***p < 0.001, ****p < 0.0001)

## 4 Discussion

In this study, single‐cell RNA sequencing of LUAD bone metastases was leveraged to identify a functionally specialized MSC C1 subpopulation, which exhibited stem‐like properties, extensive intercellular crosstalk, and strong clinical relevance. Integration of scRNA‐seq data with bulk transcriptomic and drug response data across multiple cohorts further revealed that the prognostic model developed based on this stromal subset can stratify patients into high‐ and low‐risk groups with distinct immune microenvironmental profiles, predicted chemosensitivity, and OS. This finding highlights tumor‐educated MSCs as active regulators of the metastatic niche rather than merely structural components, suggesting their potential involvement in disease progression and therapeutic response.

The trajectory analysis showed that MSCs in the bone metastatic niche undergo bifurcated differentiation toward either a matrix‐remodeling or a secretory fate (Marjanovic et al. 2020). The C1 subpopulation, localized near the root of the pseudotime trajectory and characterized by high CytoTRACE‐derived stemness scores, represented an early, less‐differentiated state (Yao et al. 2024). This finding is consistent with emerging evidence that undifferentiated stromal cells within metastatic sites retain greater plasticity and pro‐tumorigenic potential (Zheng et al. 2022; Graham and Qian 2018). Notably, C1 cells exhibited bidirectional communication with both immune and vascular compartments, transmitting signals via signaling pathways involving TGFβ, VEGFA, and WNT that may contribute to angiogenesis and immunosuppression (Hou et al. 2024; Zhou et al. 2022), while receiving inflammatory signals, such as MIF‐ACKR3, from lymphoid and myeloid cells (Liang et al. 2024). These reciprocal interactions suggest that C1 MSCs may function as integrators of microenvironmental signals, dynamically shaping a permissive niche that facilitates tumor persistence (Frisbie et al. 2022).

The prognostic model based on C1‐specific markers exhibited robust performance in both the TCGA‐LUAD training cohort and the independent GSE31210 validation set. Importantly, high RiskScores were consistently associated with reduced infiltration of adaptive immune cells, including activated B cells, CD8^+^ T cells, and effector memory subsets, indicating an immunologically “cold” tumor phenotype (Zhang et al. 2024; Poggi et al. 2018). This finding supports the notion that stromal‐driven immunosuppression contributes to poor outcomes in LUAD (Slama et al. 2023; Lu et al. 2021), consistent with observations in other solid tumors in which cancer‐associated fibroblasts or MSCs contribute to the exclusion of cytotoxic lymphocytes (Jia et al. 2025). The present findings extend this paradigm to the context of bone metastasis, highlighting the stroma as a critical determinant of anti‐tumor immunity beyond the primary site.

Notably, the prognostic model was established using MSC C1‐associated genes that collectively captured key biological processes involved in shaping the tumor microenvironment and influencing clinical outcomes in LUAD bone metastasis. Among them, *LDHA*, a key enzyme in aerobic glycolysis, exerted the strongest contribution to the RiskScore, underscoring the potential importance of metabolic reprogramming in aggressive diseases (Liu et al. 2022). Consistent with this observation, the tumor‐promoting effect of *LDHA* was validated in A549 cells. Similarly, higher expression of *PPFIBP1*, a regulator of focal adhesions (Dong et al. 2021), and *NT5E* (CD73), an immunosuppressive ectoenzyme, was associated with increased risk (Zhang et al. 2025), suggesting potential roles in enhancing metastatic adhesion and facilitating immune evasion, respectively. In contrast, higher expression of DOCK4 and NUPR1, which are involved in cytoskeletal organization and cellular stress responses (Westbrook et al. 2019; Liu and Costa 2022), was associated with lower RiskScores and more favorable prognosis, potentially reflecting effects on invasive capacity and cellular differentiation. The inclusion of *ID1*, an inhibitor of bHLH transcription factors involved in the regulation of EMT and stemness (Zhao et al. 2020), further links the prognostic signature to developmental plasticity within the metastatic niche. Collectively, these genes reflect a coordinated interplay among metabolic activation, immune modulation, cell adhesion and motility, and stress adaptation, providing a biologically grounded rationale for the predictive performance of the RiskScore.

A significant negative correlation was identified between RiskScore and the predicted IC_50_ of ERK_6604_1714, an investigational ERK pathway inhibitor. This finding suggested that patients with high RiskScores, characterized by aggressive biology and immune evasion, may exhibit increased vulnerability to targeted inhibition of MAPK signaling (Bhatt et al. 2021). Given the high prevalence of *KRAS* mutations in LUAD and their established role in driving ERK activation (Yu et al. 2025), this provides a rationale for biomarker‐enriched clinical trials of ERK inhibitors in high‐risk subgroups rather than tumor genomic alterations alone. These results underscore the potential of microenvironment‐based stratification to complement conventional molecular profiling.

Several limitations in this study should be acknowledged. First, although the scRNA‐seq dataset was related to human LUAD bone metastases, it included only ten patients, which may limit the generalizability of the findings. Moreover, both the training and validation cohorts used to construct the prognostic model consisted of unselected primary LUAD patients, and an independent cohort of patients with bone‐metastatic patients was unavailable for targeted validation. Larger multi‐center cohorts and additional bone‐metastasis datasets are therefore needed to validate MSC heterogeneity across diverse genetic and clinical backgrounds. Further clinical investigations are also warranted to confirm the reliability of the identified biomarkers. Second, ERK_6604_1714 was identified as a candidate therapeutic agent based solely on computational analyses. Further in vitro and in vivo experiments, including cellular IC_50_ assays, together with clinical studies, are required to validate its therapeutic potential and efficacy in high‐risk patients. Third, the causal role of C1 MSCs in promoting metastasis remains largely inferential. Future studies using co‐culture systems or conditional knockout models are required to establish mechanistic causality.

## 5 Conclusion

In conclusion, this study revealed that transcriptional heterogeneity within the MSC compartment contributes to functional diversification in the bone metastatic niche of LUAD. The C1 subpopulation emerged as a crucial mediator of stromal‐immune crosstalk and a source of clinically relevant biomarkers. By integrating single‐cell biology with clinical outcomes, these findings highlight the potential value of incorporating stromal features into risk assessment and therapeutic decision‐making for LUAD patients with bone metastasis.

## Author Contributions

**Houlei Wang:** conceptualization, methodology, writing – original draft, validation, software, data curation. **Tianle Ma:** conceptualization, methodology, writing – original draft, validation, formal analysis, supervision. **Chao Jia:** validation, visualization, formal analysis, supervision, resources, writing – original draft. **Chunhui Ma:** writing – review and editing, project administration, funding acquisition, conceptualization, methodology.

## Funding

This project was supported by National Natural Science Foundation of China (82403557).

## Ethics Statement

This study was based on publicly available datasets and commercially available cell lines. Ethical approval was not required as all data were de‐identified and obtained in accordance with the respective database policies.

## Consent

The authors have nothing to report.

## Conflicts of Interest

The authors declare no conflicts of interest.

## Data Availability

The datasets generated and/or analyzed during the current study are available in the (GSE31210) repository (https://www.ncbi.nlm.nih.gov/geo/query/acc.cgi?acc=GSE31210) and (GSE266330) repository (https://www.ncbi.nlm.nih.gov/geo/query/acc.cgi?acc=GSE266330).
